# Measuring the immune system of the three‐spined stickleback – investigating natural variation by quantifying immune expression in the laboratory and the wild

**DOI:** 10.1111/1755-0998.12497

**Published:** 2015-12-21

**Authors:** Shaun Robertson, Janette E. Bradley, Andrew D. C. MacColl

**Affiliations:** ^1^ School of Life SciencesUniversity of NottinghamUniversity ParkNottinghamNG7 2RDUK

**Keywords:** gene expression, host–pathogen interactions, immunoecology, immunoregulation, parasite resistance, wild immunology

## Abstract

Current understanding of the immune system comes primarily from laboratory‐based studies. There has been substantial interest in examining how it functions in the wild, but studies have been limited by a lack of appropriate assays and study species. The three‐spined stickleback (*Gasterosteus aculeatus* L.) provides an ideal system in which to advance the study of wild immunology, but requires the development of suitable immune assays. We demonstrate that meaningful variation in the immune response of stickleback can be measured using real‐time PCR to quantify the expression of eight genes, representing the innate response and Th1‐, Th2‐ and Treg‐type adaptive responses. Assays are validated by comparing the immune expression profiles of wild and laboratory‐raised stickleback, and by examining variation across populations on North Uist, Scotland. We also compare the immune response potential of laboratory‐raised individuals from two Icelandic populations by stimulating cells in culture. Immune profiles of wild fish differed from laboratory‐raised fish from the same parental population, with immune expression patterns in the wild converging relative to those in the laboratory. Innate measures differed between wild populations, whilst the adaptive response was associated with variation in age, relative size of fish, reproductive status and *S. solidus* infection levels. Laboratory‐raised individuals from different populations showed markedly different innate immune response potential. The ability to combine studies in the laboratory and in the wild underlines the potential of this toolkit to advance our understanding of the ecological and evolutionary relevance of immune system variation in a natural setting.

## Introduction

Current knowledge of the vertebrate immune system is based largely on studies of humans and a few select model species (Maizels & Nussey 2013). These studies are often laboratory‐based, performed in highly controlled settings where variation is minimized, so that cellular and molecular immune mechanisms can be identified. Natural settings, however, where real immune systems have to operate, are characterized by genetic diversity of both hosts and parasites, and by variation in the abiotic environment. Such diversity may play an important role in the ‘natural’ function of the immune system (Lazzaro & Little 2009; Martin *et al*. 2011), but is often overlooked in purely laboratory‐based studies. Placing our current understanding of the immunological mechanisms into a real‐world context is a fundamentally important challenge in advancing our understanding of the immune system.

Studies examining immune variation in the wild have hitherto suffered from a lack of appropriate assays and model species (Fassbinder‐Orth 2014). Focus to date has primarily been on a number of rodent species (For review, see Babayan *et al*. 2011; and Pedersen & Babayan 2011) where knowledge of immune function is relatively well developed due to their close relationship to laboratory models, and a range of immune‐related assays are available. We propose the development of the three‐spined stickleback *Gasterosteus aculeatus* L. (hereafter ‘stickleback’) as a species with enormous potential for use in the study of the vertebrate immune system in a wild setting.

Sticklebacks are widely distributed across a range of habitats throughout the Northern Hemisphere, from the open ocean to freshwater lakes and streams, and show a high degree of variation across this range (Colosimo *et al*. 2005; Jones *et al*. 2012a). Individuals can be sampled from a large number of replicate populations in the wild, crosses between populations are possible, and offspring can be raised in uniform laboratory conditions and controlled infection experiments performed. Sticklebacks have a well‐characterized parasite fauna (MacColl 2009; Barber & Scharsack 2010; De Roij & MacColl 2012), and a reference genome is also available (Jones *et al*. 2012b). This unique set of characteristics has made the stickleback an excellent model in the study of evolutionary and ecological parasitology (Barber 2013), and an ideal system in which to conduct a range of further immunological‐related study.

The sticklebacks found on the island of North Uist, Scotland, represent one well‐studied system (Giles 1983; De Roij & MacColl 2012; MacColl & Aucott 2014). There is growing evidence for within‐ and between‐population variation in parasite resistance (De Roij *et al*. 2011), which probably has a genetic basis (El Nagar 2014). Two common stickleback parasites found on North Uist are the ectoparasitic trematode *Gyrodactylus arcuatus* and the cestode *Schistocephalus solidus*. Individuals infected with *G. arcuatus* in controlled conditions have a 2.5% mortality rate (M. Mahmud, S. Robertson and A. D. C. MacColl, unpublished data) and grow slower than noninfected fish (De Roij *et al*. 2011). Infection with *S. solidus* causes reduced egg production of females (Barber *et al*. 2008; Heins & Baker 2008) and reduced levels of 11‐ketotestosterone, courtship behaviour and nest building in infected males (Rushbrook & Barber 2006; Barber & Scharsack 2010; MacNab *et al*. 2011). The impact of parasitism on host fitness has been well studied, but the link between infection and a host's defence is less clear.

Research has begun to examine the underlying basis of parasite resistance in stickleback, with early focus on how changes in immune‐related cell types correspond to parasitic infection (Scharsack *et al*. 2004). The role of major histocompatibility complex (MHC) in parasite resistance has also been documented (Kurtz *et al*. 2004; Wegner *et al*. 2006), as has the relative importance of MHC diversity and genetic background in determining parasite load (Rauch *et al*. 2006). A wider overview of the immune system in stickleback is required to better understand its function in a natural setting.

Teleost fish possess components of both the innate and adaptive immune system (For review, see Magnadottir 2006; and Uribe *et al*. 2011) and may be the lowest branch of vertebrates in which these are conserved (Whyte 2007; Uribe *et al*. 2011). Inflammation at the site of infection is central to the innate response, whilst the adaptive response consists primarily of B cells, T cells and antigen presenting cells. T cells differentiate into subtypes (Th1, Th2, Th17 and Treg) which mediate a response specific to the type of challenge being faced, with each subtype having a unique combination of signals and transcription factors associated with it (for a review in mammals, see Bradley & Jackson 2008); these signalling pathways appear active in teleost fish (Laing & Hansen 2011; Toda *et al*. 2011). Measuring these different components, which arise from linked immunogenetic pathways but differ in their response to infection, can give a general overview of the function of the immune system as well as indicate the nature of the challenge being faced.

Quantitative real‐time PCR (hereafter ‘qPCR’) allows the measurement and quantification of a specific target sequence. Recent advances have made whole transcriptome sequencing more accessible, but qPCR retains the advantage of being relatively cheap, allowing a much higher level of biological replication, and primers can be designed based on *a priori* understanding of the function of the immune system (Fassbinder‐Orth 2014). qPCR has been widely used in immunology to measure the expression levels of candidate immune system genes (Jackson *et al*. 2011; Bertolotti *et al*. 2013; McWilliam *et al*. 2013). Using qPCR in stickleback gives only a single time point, due to the destructive nature of sample collection. Such data can be combined with cell culture techniques, as described in Bradley & Jackson (2008), so that it is not only possible to measure the contemporary function of the immune system, but also the ability of an individual to respond to subsequent challenge. A set of qPCR reference genes are available for stickleback (Hibbeler *et al*. 2008), and some immune system assays have previously been used (Wegner *et al*. 2006; Dittmar *et al*. 2014). Whilst this previous work contains some assays for immune system genes, there remains a lack of a set of markers which provide a cohesive summary of immune system function.

The main aim of this study was to develop a set of assays which give a general overview of the function of the immune system at the time of sampling and to develop an accompanying cell culture technique to measure the potential of an individual's immune system to respond to challenge. Previous work in our study populations has shown there to be a genetic basis to resisting parasitic infection (El Nagar 2014; M. Mahmud, S. Robertson and A. D. C. MacColl, unpublished data), so we examine whether there are underlying differences in both immune expression profiles and innate response potentials, in fish which have been raised in controlled conditions, which may play a role in this. To show that the assays presented can capture variation in the immune response, we compare immune expression profiles between laboratory‐raised and wild individuals from the same parental populations. Finally, we examine variation across six populations which contain contrasting parasite communities in order to identify factors which may contribute to shaping an individual's immune response. The techniques presented will allow this common model species to move to the forefront of wild immunological research and provide a base on which further measures can be added and refined over time.

## Methods

### Assay development

Candidate genes which represent different functional arms of the immune system were identified based on the known roles of orthologous genes and their presence in previous studies in other fish species. The innate immune system is characterized by the expression of IL‐1*β* and TNF‐*α*, which promote, and IL‐10 and TGF*β* which regulate, inflammation. Th1‐like responses are characterized by expression of IL‐12, T‐bet, Stat4 and IFN‐*γ*, whilst Th2‐like responses are characterized by Gata‐3, Stat6, CMIP and IL‐4, ‐5, IL‐9 and IL‐13. Th17‐like responses are characterized by TGF*β*, ROR‐*γ*t and IL‐6, IL‐17, IL‐22 and IL‐23, with the immunosuppressive Treg‐like response being characterized by TGF*β*, FoxP3a and IL‐10.

To get sequences for candidate genes, the stickleback genome was accessed through ENSEMBL release 77 (Flicek *et al*. 2014). If a gene was present and named, the coding sequence was downloaded. A blastx search was performed against the NCBI protein sequence database to confirm that the correct gene had been selected. Where no named gene was found, the sequence of the target gene from another fish species was used to perform a tblastx search against the stickleback genome in ENSEMBL. The sequence of any unnamed stickleback gene showing high similarity was downloaded, and a blastx search performed against the NCBI protein sequence database, to confirm the identity of the unnamed gene.

Three pairs of primers were designed for each gene using primer3web v4.0.0 (Koressaar & Remm 2007; Untergasser *et al*. 2012), using default parameters with the following modifications: primer size 18–23 bp, melting temperature 57–62 °C, GC content 30–70% and a product size of 90–150 bp. Where possible, the product spanned an exon or one primer crossed an exon:intron boundary to avoid dsDNA amplification. A blastn search was performed against the NCBI nucleotide sequence library to ensure primer specificity to the gene of interest.

### Primer screening

Initial screening for amplification was performed on pooled cDNA samples from multiple individuals, caught on North Uist in Spring 2013 (methods as described below), with each primer at a 2 *μ* m concentration. Primers were retained for further testing if logarithmic stage amplification occurred before 40 cycles. Efficiency tests were performed on fivefold serial dilutions of cDNA from single individuals in triplicate replicates. Efficiency values were calculated, with a desired range between 75% and 110% and an *R* ^2^ value > 0.98. The average efficiency of primers fulfilling these criteria was calculated across 3 separate efficiency runs. If multiple primers for each gene reached this stage, the best pair was selected based on amplification plots, efficiency % and *R* ^2^ values. Primers which failed to meet the criteria were tested at a range of concentrations (2, 3, 4 and 6 *μ* m) and retained if they met the specification at a new concentration.

### Primer validation

The repeatability level of each primer pair was determined as the lowest cDNA concentration at which amplification was detected across triplicate replicates in the three efficiency runs. To confirm the specificity of amplification, PCR product from each primer pair was sequenced. A PCR was performed on a pooled cDNA sample in a 20 *μ*L total volume, containing 10 *μ*L BioMix Red (Bioline), 6 *μ*L H_2_O, 1 *μ*L of each 10 *μ* m primer and 2 *μ*L template cDNA. An initial step of 4 min at 95 °C was followed by 35 cycles of 95 °C for 15 s, 58 °C for 15 s and 72 °C for 30 s, with a final extension of 72 °C for 4 min. Amplification was confirmed by visualization of 5 *μ*L of product on a 2% agarose gel stained with ethidium bromide. Samples with positive amplification were Sanger sequenced in both forward and reverse orientation (Source Bioscience, Nottingham, UK). The sequences for each gene were aligned with the reference and primer sequences to ensure the correct amplicon was present.

### Sample collection

For the examination of immune profiles in wild fish, individuals were sampled from populations on the island of North Uist, Scotland, in October 2014. Fish were caught in Gee's Minnow Traps, set overnight for approximately 16 h in six lochs (Table 1) which contain contrasting parasite communities. All individuals caught in a population were pooled, and a subset was randomly selected for inclusion in this study. Live fish were transported directly to the laboratory in darkened boxes containing water from their population, with air from battery powered pumps, and processed in a haphazard order within 3 h of collection. No correlations were found between gene expression and sampling order (see Appendix S1 and S2, Supporting information), indicating that holding time had no significant effect on individual immune expression levels.

**Table 1:** North Uist wild stickleback populations sampled in October 2014, including prevalence and intensity of the two main parasite species found

Loch	Short name	Location	*n*	*Gyrodactylus*		*Schistocephalus*	
				Prevalence	Intensity	Prevalence	Intensity
a'Bharpa	Bhar	57°34′20″N; 7°18′11″W	15	0 (0–21.8)	0	60 (32.3–83.7)	1 (± 0)
Hosta	Host	57°37′30″N; 7°29′8″W	16	68.8 (41.3–88.9)	2.55 (± 0.53)	43.8 (13.8–70.1)	2.14 (± 0.51)
Nam Magarlan	Maga	57°36′15″N; 7°28′51″W	15	53.3 (26.6–78.7)	3.38 (± 0.68)	20 (4.3–48.1)	1 (± 0)
Unnamed	Muay	57°36′23″N; 7°10′38″W	15	40 (16.3–67.7)	1.83 (± 0.40)	0 (0–21.8)	0
Ob nan Stearnain	Obse	57°36′9″N; 7°10′19″W	15	46.7 (21.3–73.4)	1.43 (± 0.20)	0 (0–21.8)	0
Reivil	Reiv	57°36′38″N; 7°30′49″W	16	56.3 (33.3–80.3)	2.44 (± 0.41)	0 (0–20.6)	0

‘n’ indicated the number of individuals sampled. Prevalence was calculated as the percentage of individuals infected, with 95% binomial confidence intervals in brackets. Intensity data are mean number of parasites on infected individuals, with standard errors.

Fish were euthanized using the appropriate schedule 1 technique according to UK Home Office regulations (overdose of MS‐222 followed by destruction of the brain), then measured and weighed, and their spleens, an immunologically important tissue in fish (Zapata *et al*. 2006), removed and immediately placed in RNAlater (Life Technologies). Spleen samples were kept at 4 °C for 24 h, then at −20 °C for no more than 6 months until RNA extraction. Sex was recorded along with reproductive status, defined as whether there was evidence of investment in attaining reproductive condition, determined by visual examination of the gonads. Males were deemed to be reproductive if their testes and kidneys were enlarged, whilst females were deemed to be reproductive if any ovules were enlarged.

Macroparasites were identified and counted under a dissection microscope. Where individuals were infected with *S. solidus*, the total weight of all plerocercoids (the life stage infecting stickleback) was recorded, and the parasite index calculated as the total proportion of a fish's body weight accounted for by the parasite (Arme & Owen 1967). An individual's condition was calculated using Fulton's condition factor, *K* = 10^5^ × Weight (g)/Length (mm)^3^ (Cited in Nash *et al*. 2006), with a high *K* value indicating good condition. Calculations for individuals infected with *S. solidus* were adjusted using individual weight minus the total weight of all plerocercoids. Age of fish was determined by examination of the annuli in sagittal otoliths (Jones & Hynes 1950).

To create families of laboratory‐raised fish, adults in breeding condition were caught in two lochs, Reiv and Obse (Table 1) in Spring 2013. Fish were crossed to create families following the protocol in Leinonen *et al*. (2011). Fertilized eggs were transported to an aquarium at the University of Nottingham, and after hatching, families were raised in 25 L partitions of 100‐L tanks under uniform environmental conditions. Single fish from each of six families from Reiv and six families from Obse were euthanized when 11 months old, using the appropriate schedule 1 technique according to UK Home Office regulations. Their spleens were removed and immediately placed in RNAlater (Life Technologies) and kept at 4 °C for 24 h, then stored at −20 °C for no longer than 6 months until RNA extraction was performed.

To compare innate immune responsiveness between populations, adults in breeding condition were caught from Holsvatn (64°04′N 21°52′W, hereafter ‘Hols’) and Vífilsstaðavatn (54°31′N 22°08′W, ‘Vifi’) in Iceland in June 2014. Crosses were made between adults in breeding condition to create families following the approach of Leinonen *et al*. (2011). Fertilized eggs were transported to the University of Nottingham and raised as above. Single individuals from each of 17 families from Hols and 20 families from Vifi were euthanized in March 2015, using the appropriate schedule 1 technique according to UK Home Office regulations, then measured and weighed. Head kidneys were removed and immediately stored in ice cold RPMI 1640 supplemented with 10% H_2_O for use in cell culture.

### Primary culture of stickleback cells

A full step‐by‐step cell culture protocol can be found in the Appendix S1 and S2 (Supporting information), adapted from the protocol of Hibbeler *et al*. (2008). Briefly, head kidneys were disaggregated through a 40‐*μ*m cell strainer into RPMI 1640 supplemented with 10% H_2_O. Cells were washed and four replicates per fish were cultured (20 °C, 5% CO_2_) on 96‐well plates at 2 × 10^6^ cells/mL in 100 *μ*L of culture media, containing RPMI 1640 supplemented with 10% H_2_O; 10% heat‐inactivated foetal calf serum; 2 mm l‐glutamine; 100 *μ*g/mL penicillin; and 100 *μ*g/mL streptomycin. Two replicates per fish served as a control whilst the others were stimulated with 12.5 *μ*g/mL zymosan (Sigma‐Aldrich Z4250), a general antagonist of the innate immune response. After 24 h, cells were removed from culture, the supernatant was removed, and cells were stored at −80 °C until RNA extraction was performed.

### Sample preparation and reaction conditions

All qPCR work was performed in accordance with the MIQE guidelines (Bustin *et al*. 2009; Taylor *et al*. 2010). RNA was extracted from whole spleens using the GeneJET RNA purification kit (Thermo Scientific) according to the manufacturers’ protocol. Extractions from cultured cells were performed using the RNAqueous‐Micro total RNA isolation kit (Ambion AM1931) following the manufacturers’ protocol.

Purity of RNA was assessed on a NanoDrop 1000 spectrophotometer (Thermo Scientific), with a desired 260/280 absorbance ratio > 1.80. RNA integrity was assessed by incubating 5 *μ*L of each sample at 65 °C for 10 min, followed by visualization on a 2% agarose gel stained with ethidium bromide. All samples were DNase treated using Precision DNase (Primer Design), following the manufacturers’ protocol. Reverse transcription reactions were performed on approximately 1.5 *μ*g of total RNA using the nanoScript2 RT kit (Primer Design), with a combination of random nanomer and oligo‐dT priming, following the manufacturers’ protocol. Periodic no‐enzyme controls indicated that genomic DNA contamination was negligible. All cDNA samples were diluted 1:10 with nuclease‐free water before further use.

qPCRs were performed in 10 *μ*L total volumes, containing 5 *μ*L of PrecisionFAST mastermix with SYBR green (Primer Design), 0.25 *μ*L of each primer at working concentration (see Table 2), 2 *μ*L of template cDNA and 2.5 *μ*L of H_2_O in 96‐well optical PCR plates with optical seals (StarLab). Reactions were performed in an ABI 7500 FAST real‐time thermocycler (Applied Biosystems) at 95 °C for 20 s, followed by 45 cycles of 95 °C for 3 s and 60 °C for 30 s. All runs included a post‐PCR melt curve analysis.

**Table 2:** Primer details for 8 immune system assays in three‐spined sticklebacks, including working concentrations and average PCR efficiency values

Gene name	ENSEMBL ID	Primer Sequence (5′ – 3′)	Amplicon length	Working Conc	PCR Eff	Repeatability level
IL‐1*β*	ENSGACG00000014611	Fwd‐ACGGCTCGGAGTTGCTGAT	100	2	85	0.4
		Rev‐CTGCACAGCGTCACGATCTC				
TNF‐*α*	ENSGACG00000013372	Fwd‐GCTTGGTTCTGGCCAGGTTT	125	2	87.9	0.4
		Rev‐GCTGCTGATTTGCCTCAACG				
TGF*β*	ENSGACG00000012798	Fwd‐TCCCGCTTCGTCACCAACCA	140	2	96.5	0.08
		Rev‐ACGTCTGTCTGGCCACATTCAC				
Stat4	ENSGACG00000002684	Fwd‐CTCTCAGTTTCGAGGCTTGCTT	100	2	93.5	0.016
		Rev‐GGCAGTTGGCTCACATTGG				
T‐bet	ENSGACG00000003829	Fwd‐CACATCGTGGAGGTGAAGGA	99	4	79.8	0.08
		Rev‐CGGTGACGGCGATGAACT				
Stat6	ENSGACG00000008477	Fwd‐CTCAGCCACAGTTCCAACCGTTC	104	3	100.9	0.08
		Rev‐GTCGGATGTTCTGGACCTCGAGT				
CMIP	ENSGACG00000002527	Fwd‐GGCATGGAGGTCGTCAAGAA	119	3	100.6	0.08
		Rev‐TAGCAGGAGTAAATGGCGGC				
FoxP3a	ENSGACG00000012777	Fwd‐GTTGACCCATGCAATTCCGA	94	2	98.9	0.4
		Rev‐CTGCTGTAGTTGTGGTCCTG				

‘ENSEMBL ID’ data give the gene on which primer designs were based. ‘Amplicon Length’ indicates the length of product in base pairs. ‘Working Conc’ gives the working concentration of each primer in *μ* m, with 0.25 *μ*L each added to a 10 *μ*L total reaction volume. ‘PCR Eff’ gives the efficiency of amplification for each primer pair, in %, averaged over 3 runs with *R* ^2^ > 0.98. ‘Repeatability Level’ indicates the lowest total cDNA concentration, in ng/*μ*L, at which amplification was consistently detected across triplicate replicates in three efficiency runs.

### Gene expression quantification

Accurate normalization is essential for the production of reliable data from qPCR experiments, with the optimal reference genes being specific to a particular set of experimental conditions (Dheda *et al*. 2005). To select the most appropriate reference genes for this study, a geNorm analysis was performed on six candidate reference genes (B2M, GAPDH, RPL13A, HPRT1, TBP and TOP1) using a custom stickleback geNorm kit for SYBR green (Primer Design), following the manufacturers’ standard protocol. For the wild expression study, 12 spleen cDNA samples from wild fish were included, comprising two randomly selected individuals from each study population. Analysis of the stability of expression was performed in qbase+ (Biogazelle), with B2M and RPL13A identified as the most stable combination of reference genes. TBP and HPRT1 were identified as the optimum combination for the cell culture samples, after stability analysis on 7 random samples from Hols and 7 from Vifi.

To quantify immune gene expression in wild fish, a total of 92 samples were used. Numbers contributed by each population are shown in Table 1, plus 12 samples from laboratory‐raised control fish. A reference sample was made by pooling cDNA from all experimental samples, to control for between‐plate variation. Samples were randomly split between plates, with each plate also containing the reference sample and negative controls. Reactions were performed in duplicate for each sample.

A total of 37 cell culture cDNA samples were included for expression measurements of IL1*β*, TNF‐*α* and two reference genes. A reference sample was made by pooling cDNA from all samples. Reactions were performed in duplicate for each sample, and all plates included the reference sample and negative controls.

Relative expression values were calculated using the ΔΔCq method (Pfaffl 2001), adjusted for the amplification efficiencies of each primer pair and standardized against the geometric mean Cq of the two reference genes for each sample (Vandesompele *et al*. 2002). For cell culture data, the relative increase in expression in zymosan‐stimulated vs. unstimulated control cells was calculated for both genes.

### Data analysis

All data were log_10_‐transformed prior to analysis, due the inherently skewed distribution of relative expression data. Analysis was performed in r v.3.1.2 (R Core Team 2014).

#### Laboratory vs. wild comparisons

In the first instance, expression profiles of the eight immune genes were compared between wild and laboratory‐raised fish for pairs of populations (Reiv and Obse) for which both were available. Multivariate analysis of variance (manova) was used to test for differences between Reiv and Obse laboratory bred fish, Reiv laboratory and wild fish, and Obse laboratory and wild fish. Overall differences were assessed using the Pillai method, followed by examination of each gene separately. To simultaneously summarize the expression profiles of the groups in multivariate space, a principal components analysis (PCA) was performed on immune expression measures for all groups, using the ‘prcomp’ function on the variance–covariance matrix, and individual scores for the first and second principal components were plotted. To compare the separation of each group in multivariate space, the average Euclidean distance (± SE) was calculated between each group, based on the expression values of all immune genes.

#### Expression profiles in wild populations

To examine which factors related to the expression of immune genes in the sampled wild populations, PCA was followed by the fitting of general linear models containing recorded factors which we expected to influence gene expression measurements. PCA is a widely used data reduction technique which converts a number of positively correlated variables into a smaller set of principal components which summarize the variation present in the original measures. In this way, multiple expression measurements of functionally related components of the immune system can be summarized by a single measure, composed of the individual scores from a dominant first principal component (PC1), following the approach previously employed by Jackson *et al*. (2011). Measures were split into four functional groups based on their role: IL‐1*β* and TNF‐*α* (innate); Stat4 and T‐bet (Th1‐like); Stat6 and CMIP (Th2‐like); and FoxP3a and TGF*β* (immunosuppressive).

PCA was performed using the ‘prcomp’ function, based on the variance–covariance matrix, with individual PC1 scores, then used in subsequent analysis. FoxP3a and TGF*β* expressions were not positively correlated, so they were subsequently analysed separately. For other PCAs, the first component explained a high proportion of variation in the data (>60%) and both factors contributed positively to the loadings.

General linear models (GLMs) were fitted using PC1 score, or FoxP3a and TGF*β* expression measures, as the response variable. Maximal models contained Population (6 levels), Sex (2 levels), Reproductive Status (2 levels) and *G. arcuatus* Presence (2 levels) as factors, and Adjusted Length (calculated as individual length minus population average length), *S. solidus* Parasite Index, Age and Condition Score as continuous variables. Biologically relevant interaction terms were also fitted. Nonsignificant terms were sequentially dropped to generate a minimum adequate model. Significance of remaining components was determined by Wald *F*‐tests.

*Gyrodactylus arcuatus* and *S. solidus* were the dominant parasite species found across populations, so the only parasites included in the full models. In one population, Obse, lice of the family Argulidae were found in high prevalence. To investigate their effect within the population, a model was fitted to Obse Innate, Th1, Th2, FoxP3a and TGF*β* measures separately, with the addition of Lice Count as a continuous variable replacing the *S. solidus* Parasite Index term and interactions.

#### *In vitro* response potential

The difference in response potential of fish from Hols and Vifi was examined by comparing the change in expression of IL‐1*β* and TNF‐*α* in stimulated vs. control cultured cells. The distribution of expression values for both genes was not normal (Shapiro–Wilk test, results not shown), so the difference in responsiveness between populations for each gene was compared using Mann–Whitney U‐tests.

## Results

### Assay development

Initially 22 potential candidate genes were identified, of which 16 were present in the stickleback genome. Primers for 12 genes showed consistent amplification across a number of cDNA samples (for full details of targeted genes, see Table S1, Supporting inofrmation). A total of eight genes met the required amplification criteria, with IL‐1*β*, TNF‐*α*, TGF*β*, Stat4 and FoxP3a optimized at 2 *μ* m, and Stat6, CMIP and T‐bet optimized at higher concentrations (Table 2). Primer efficiencies ranged from 79.8% to 100.9%, with repeatability levels of 0.4–0.016 ng/*μ*L of template cDNA. For each primer pair, PCR product was successfully sequenced and aligned back to the expected location, with blastx searches against the NCBI protein sequence database further confirming that the intended targets were amplified.

### Sampling wild populations

*Gyrodactylus arcuatus* and *S. solidus* were found to be the dominant parasites across the wild populations sampled (Table 1). The prevalence of *G. arcuatus* differed significantly between populations ($\chi_{5}^{2}$ = 17.4, *P* = 0.004), as did the prevalence of *S. solidus* ($\chi_{5}^{2}$ = 31.4, *P* < 0.001). Lice of the Argulidae family were found only in Obse, with a prevalence of 86.7% (95% binomial confidence interval 59.5–98.3%), and an average intensity on infected individuals of 2.92 (SE ± 0.84). No other parasite species were identified in large numbers. All populations contained both male and female fish, with individuals of both sexes varying in their reproductive condition.

### Laboratory vs. wild comparisons

**Figure 1:**
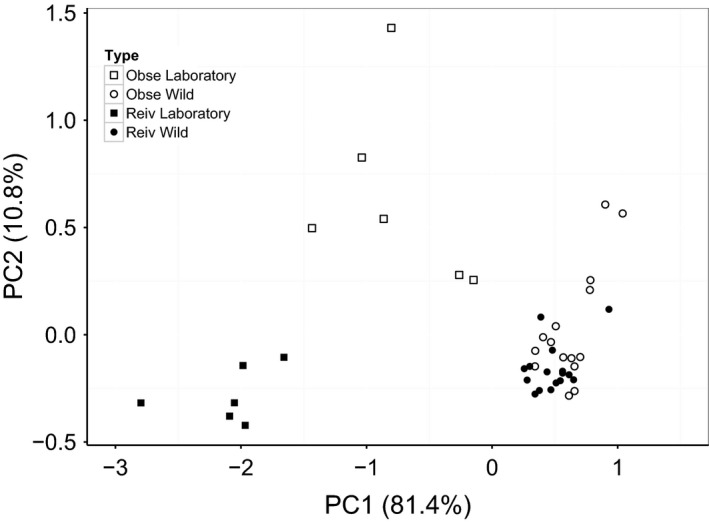
Plot of the first two principal component axes (PC1 and PC2) from a principal components analysis of the expression profiles of eight immune genes, with numbers in brackets indicating the percentage of variation explained in each axis. Fish are grouped by type, indicating the population from where they came (‘Obse’ or ‘Reiv’) and whether they were caught in the wild (‘Wild’) or laboratory raised (‘Laboratory’).

There was a significant difference in the immune profiles of laboratory‐raised fish from Reiv and Obse (manova *F*_1,10_ = 16.92, *P* = 0.020), primarily driven by differences in innate, Th2‐type and immunosuppressive measures. Wild fish from Reiv differed from Reiv laboratory fish (manova *F*_1,20_ = 11.31, *P* < 0.001), with wild fish having higher expression of all genes. Wild fish from Obse also differed from Obse laboratory fish (manova *F*_1,19_ = 4.40, *P* = 0.011), primarily driven by higher expression of Th1‐type genes in wild fish. Full details of comparisons are shown in Table S2 (Supporting information). PCA on all groups found a PC1 which explained 81.4% of variation in expression, with a high PC1 score indicating high expression of all immune genes. The PC2 axis explained 10.8% of variation, with a high score indicating high expression of IL‐1*β* and TNF‐*α*, and low expression of Stat4 and T‐bet. Laboratory‐raised fish were widely separated in the PCA (Fig. 1, average Euclidean distance 1.797 ± 0.076), whilst wild fish were not (0.534 ± 0.018). Reiv laboratory and wild fish were closer in expression profile (2.601 ± 0.039) than Obse laboratory and wild fish (1.701 ± 0.054).

### Expression profiles in wild populations

**Figure 2:**
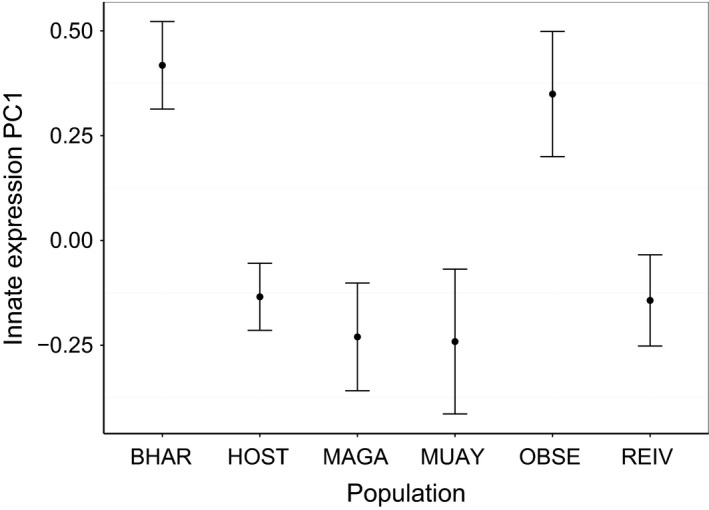
Innate immune expression scores (Mean PC1 ± SE) for each study population. A high PC1 score indicates high expression levels of IL‐1*β* and TNF‐*α*.

A summary of significant components in fitted models is shown in Table 3. Across all populations of wild fish, PC1 of innate measures explained 86.4% of variation in the data, with loadings of IL‐1*β *= 0.987 and TNF‐*α *= 0.157. A high PC1 score indicates a high level of expression, with populations differing in their expression (Fig. 2, *F*_5,85_ = 4.97, *P* = 0.002).

**Table 3:** Summaries of minimum adequate GLMs fitted to expression data of genes representing different functional arms of the immune system from wild fish caught on North Uist, Scotland. ‘Innate’, ‘Th1’ and ‘Th2’ response variables are first principal components of the following pairs of genes, respectively: IL‐1*β* and TNF‐*α*; Stat4 and T‐bet; Stat6 and CMIP

System	Factor	*F*	d.f.	*P*
Innate	Population	4.97	5,85	**0.002**
Th1	Adjusted Length	7.19	1,87	**0.009**
	*Schistocephalus solidus* index	6.22	1,87	**0.015**
	Condition (K)	3.89	1,87	0.052
	Condition (K): *S. solidus* index	5.18	1,87	**0.025**
Th2	Age	3.75	2,84	**0.028**
	Condition (K)	2.31	1,86	0.132
	Age: Condition (K)	3.55	2,84	**0.033**
FoxP3a	Adjusted Length	1.17	1,84	0.283
	Repro. Score	0.01	1,81	0.912
	Age	0.44	2,81	0.648
	*S. solidus* Index	0.01	1,84	0.905
	Adjusted Length: Age	5.42	2,81	**0.006**
	Repro. Score: *S. solidus* Index	6.24	1,81	**0.015**
TGF*β*	*S. solidus* Index	10.25	1,90	**0.002**

Values in bold indicate significant *P*‐values (*P* < 0.05).

**Figure 3:**
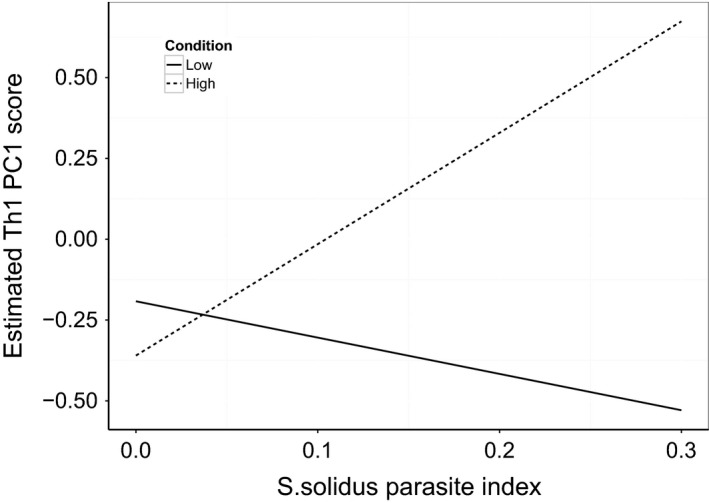
The effect of *Schistocephalus solidus* parasite index on Th1 PC1 score is dependent upon condition. A high Th1 PC1 score indicates high expression levels of Stat4 and T‐bet. Lines are estimates calculated from the fitted model, for the lowest (‘Low’, *K* = 0.75) and highest (‘High’, *K *= 1.40) Fulton's condition scores found in the sampled individuals.

The Th1‐like measures had a PC1 accounting for 62.4% of variation, with loadings of Stat4 = 0.802 and T‐bet = 0.597. Longer fish in each population had higher expression levels (*F*_1,87_ = 7.19, *P* = 0.009). An increasing *S. solidus* parasite index was associated with a decrease in expression (*F*_1,87_ = 6.22, *P* = 0.015), but this affect also varied with condition (Fig. 3, *F*_1,87_ = 5.18, *P* = 0.025), such that in uninfected fish expression decreased with increasing condition, but in heavily infected fish expression increased with condition. PC1 of Th2‐like measures explained 83% of variation, with loadings of CMIP = 0.870 and Stat6 = 0.241. Individuals in their third year (2+) had lower Th2‐like expression levels than those in their first (0+) or second (1+) (*F*_2,84_ = 3.75, *P *= 0.028). This affect also varied with condition (*F*_2,84_ = 3.55, *P* = 0.033), with individuals in good condition having lower expression when 0+ and 1+, but higher expression when 2+.

**Figure 4:**
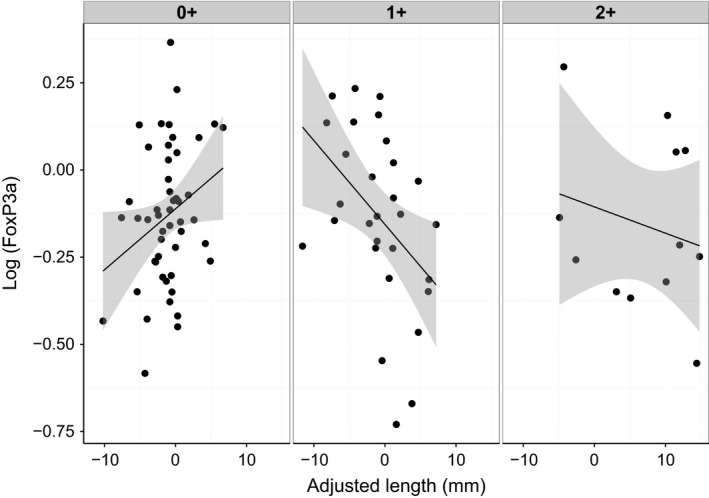
The relationship between the size of an individual, relative to the average for its population, with FoxP3a expression levels changes with age. Each box contains fish of a different age (0+, 1+, or 2+), and shaded areas indicate 95% confidence levels for fitted lines. At 0+, expression increases with age, whilst the opposite pattern is seen in 1+, with no significant change in 2+.

Adjusted length had an effect on FoxP3a expression which was dependent upon age (Fig. 4, *F*_2,81_ = 5.42, *P* = 0.006), with relatively large individuals having higher expression when 0+, but lower expression when 1+. There was an effect of *S. solidus* parasite index dependent upon reproductive condition (*F*_1,81_ = 6.24, *P* = 0.015), with no effect of *S. solidus* parasite index in reproductive individuals, but a decrease in expression with increasing parasite index in nonreproductive individuals. TGF*β* expression levels increased with increasing *S. solidus* parasite index (*F*_1,89_ = 9.84, *P* = 0.002).

When Obse data were analysed separately, no factors were found to be significant in models for Th1‐like or Th2‐like expression. Innate measures were summarized by a PC1 accounting for 78.7% of variation, with contributions of IL‐1*β* = 0.999 and TNF‐*α* = 0.019. Males were found to have higher expression levels than females (*F*_1,7_ = 6.414, *P* = 0.039) and expression increased with condition (*F*_1,7_ = 10.463, *P* = 0.014). The presence of *G. arcuatus* was associated with an increase in expression (*F*_1,7_ = 6.101, *P* = 0.042), whilst increasing lice numbers were associated with a decrease (*F*_1,7_ = 8.366, *P* = 0.023). The relationship between size and expression varied with *G. arcuatus* presence (*F*_1,7_ = 15.594, *P* = 0.006), with uninfected fish showing a decrease in expression with increasing length, and infected fish showing an increase. The effect of size also varied with lice number (*F*_1,7_ = 12.376, *P* = 0.010), with an increase in length corresponding to a more negative relationship with expression when lice number increased. Individuals infected with *G. arcuatus* had lower FoxP3a expression levels (*F*_2,13_ = 6.324, *P* = 0.012). An increase in lice was associated with an increase in TGF*β* expression levels (*F*_1,11_ = 4.89, *P* = 0.049), and this affect was also dependent upon age (*F*_1,11_ = 5.75, *P* = 0.035), with less of an increase seen in older individuals.

### *In vitro* response potential

**Figure 5:**
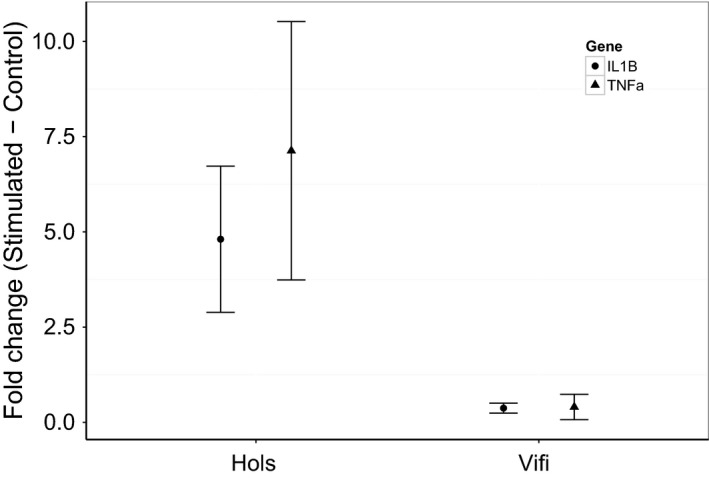
The relative fold change (stimulated minus control) in expression levels (Mean ± SE) of two innate immune system genes in cultured head kidney cells from laboratory‐raised fish from two populations. Fish from Hols show higher responsiveness in both genes than those from Vifi.

Innate immune response potential, measured as the increase in gene expression in stimulated vs. unstimulated cultured cells, was higher in individuals from Hols than those from Vifi (Fig. 5) for both IL‐1*β* (W = 208, *P* = 0.009) and TNF‐*α* (W = 138, *P* = 0.018). The average increase in relative expression of IL‐1*β* was 4.81 (SEM ± 1.92) in Hols, but only 0.37 (± 0.13) in Vifi. The increase in relative expression of TNF‐*α* was 7.13 (± 3.34) in Hols and 0.40 (± 0.33) in Vifi.

## Discussion

Laboratory‐raised fish from alternate parental populations, kept in controlled conditions with no previous pathogen exposure, differ in both their immune response potential and immune expression profiles. Fish from different wild populations show contrasting immune expression profiles, and surveying multiple populations has identified a range of factors which may be involved in shaping the immune response. The suite of qPCR assays and accompanying cell culture technique developed has allowed us to study variation in the immune response in both the laboratory and the wild, and represents a significant advance in our ability to study immunology in a natural setting since hitherto the number of wild species with such a toolbox has remained small, and been largely confined to rodents. By selecting markers which represent different response types, and which respond to contrasting types of challenge, it is possible to get a general overview of an individual's immune phenotype. Our results were consistent with expectations in several respects, giving us confidence that they are meaningful and useful. It is hoped these tools will facilitate the use of stickleback to study a range of topics relating to the function of the immune system, with the addition of further assays allowing the panel to grow and refine over time.

Laboratory‐raised fish from Reiv and Obse showed significant differences in immune profiles. As these fish have been raised and maintained in uniform laboratory conditions with no exposure to any parasites, there appears to be some genetic basis for determining an individual's baseline immune profile. Comparisons between wild fish and laboratory individuals from the same parental population showed differences in immune profiles. Fish from Reiv showed higher expression of all genes in the wild, whilst wild fish from Obse showed significantly higher Th1‐type expression compared to their laboratory counterparts. Both PCA analysis and Euclidean distances show that the expression profiles of wild fish converge relative to their laboratory‐raised counterparts (Fig. 1). Conditions encountered in the wild appear to have a larger effect on shaping the immune profile than the underlying genetic differences controlling baseline immune expression, supporting previous work showing that immune expression profiles of stickleback converge during reciprocal transplant experiments (Stutz *et al*. 2015). The increase in immune expression seen in the wild relative to laboratory‐raised fish suggests that the immune system is being stimulated by a range of possible factors in the wild. For example, Obse fish have consistently high Th1‐type expression, suggesting their immune system may be primed to respond to intracellular pathogens. Further studies are required to see whether the possible genetic control of baseline expression is present across a larger number of populations and to examine the link between this baseline expression and the common immune system antagonists present in the wild.

Wild individuals showed a high degree of variation in immune gene expression, both within and between populations, with a range of factors relating to the measured responses. The sampled populations differed in innate expression levels, but not in adaptive immune measures. The innate immune system responds rapidly to contemporary threats, whilst the adaptive response develops over a longer period to continued challenge; differences in innate immune response accounted for by the population term in the model may be due to additional within‐population factors that were not measured in this study. Lochs may differ in environmental factors, such as temperature, pH and salinity (Giles 1983; MacColl *et al*. 2013), infection levels of microparasites, and stress levels, all of which may affect the innate immune response (Castillo *et al*. 2009; Magnadottir 2010). Expression of the adaptive response appears to be better explained by factors which exist above population level variation.

A complex range of factors were found to shape an individual's immune profile within a single population. When fish from Obse were analysed alone, due to the presence of an ectoparasite not found in other populations, a number of factors, including sex, condition and the presence of *G. arcuatus* and lice, were found to be significantly correlated with innate immune expression levels. Due to the immediate and rapid nature of the innate immune system, it would be predicted that an individual's response will be shaped by the set of conditions currently being experienced at the time of sampling. Previous studies of Soay sheep (Nussey *et al*. 2014) and field voles (Jackson *et al*. 2011) find similar complex sets of characters to be involved in shaping the immune response within single wild populations. Whilst studies in stickleback provide the ability to look for general patterns across multiple replicate populations, these findings reiterate the importance of not overlooking subtle within‐population patterns which may exist.

Age was found to relate to the expression of the Th2‐type response and of FoxP3a. 2+‐year‐old fish showed lower expression of Th2 markers than 0+‐ or 1+‐ year olds. FoxP3a was associated with an age by adjusted length interaction (Fig. 3), with larger individuals having higher expression in 0+ aged fish, whilst the reverse pattern was seen in 1+ fish. Changes in Th2 and FoxP3a expression levels could be the result of a decline in immune potential with age, a pattern of growing interest in immunological research (Aw *et al*. 2007; Vleck *et al*. 2011). This pattern could also represent the transition from a resistant to tolerant immune strategy, as has been observed in wild populations of field voles (Jackson *et al*. 2014) and Soay sheep (Hayward *et al*. 2014). Further studies are required to examine whether tolerant and resistant phenotypes exist in stickleback and to further examine the changes in response which occur with age.

Infection with *S. solidus* was associated with changes in Th1‐type, TGF*β* and FoxP3a expression levels. Changes in the function of the immune system with *S. solidus* infection have previously been observed (e.g. Scharsack *et al*. 2004, 2007), with growing evidence that there is parasite‐driven manipulation of the host immune system (for summary, see Barber & Scharsack 2010). To date, there has been limited understanding of the role of Th1/Th2 immunity in this context, primarily due to a lack of appropriate assays. In schistosome infections in mice, hosts are driven towards the Th1‐type arm, so the parasite can avoid potentially harmful humoral responses (Herve *et al*. 2003). Fish in good condition had higher Th1‐type expression when infected, which could be a result of parasites manipulation if driving the host to a Th1‐type response decreases the potentially harmful Th2‐type response. Possible patterns of host manipulation warrant further investigation; the assays present provide an ideal toolkit to investigate the Th1/Th2 trade‐off in this context and to shed new light on the role of *S. solidus* in manipulating the host immune system.

Fish from Hols showed higher innate immune responsiveness than those from Vifi. These fish were raised in common garden conditions, so differences in their ability to respond to challenge are likely to have a genetic basis. These results are consistent with previous work examining the respiratory burst response of different stickleback populations to varying parasite antigens (Franke *et al*. 2014). The high responsiveness of Hols fish could suggest they have evolved in an environment which requires a large, rapid innate response. Further studies which examine innate immune responsiveness across multiple populations could identify general patterns which shape response potential. Antagonists are available for other arms of the immune system (see Jackson *et al*. 2011), which will allow a similar approach to be taken for other immune response types.

The assays developed represent both the innate and adaptive responses, and give a general overview of different functional arms of the immune system. A number of other genes were identified for which working primers could not be developed, which may be a result of sequence divergence between fish from North Uist and the stickleback reference genome (a fish from Bear Paw Lake, Alaska). Genes of the immune system have been shown to undergo higher levels of selection across a range of taxa (Nielsen *et al*. 2005; McTaggart *et al*. 2012); thus, some immune genes may show high degrees of sequence divergence between two isolated lineages within a species. Combining new sequences from European stickleback with the reference genome should allow primers which function across a wide geographic range to be designed for more genes, thus allowing assays to be added and refined over time.

The primers developed were found to be reliable and repeatable, with amplification efficiencies all within the desired range of 75–110%. *R* ^2^ values > 0.98 indicate a low degree of variability at each concentration tested. Primers amplified across a range of cDNA concentrations, from 10 ng/*μ*L to between 0.4 and 0.016 ng/*μ*L. The actual concentration of target transcript in these samples is likely to be much lower, as the measured concentrations only indicate the total cDNA added to each reaction and not the concentration of template of interest. The primers used are sensitive to a wide range of template concentrations, allowing accurate quantification across varying expression levels. The primers have successfully amplified cDNA from fish from Scotland, England and Iceland, showing that they function across a wide geographic range.

## Conclusions

Differences in immune expression profiles and response potential between laboratory‐raised fish show that there is a genetic basis controlling an individuals’ baseline immune function and response potential, possibly reflecting the need for a more robust response in certain populations. Profiles in wild populations converge relative to their laboratory‐raised counterparts, with the conditions faced by an individual having a greater effect on shaping the immune response than genetic background. Variation in expression levels of different functional components in wild populations was related to a range of factors, including size, age and *S. solidus* infection levels. Measures of the innate response reflect the current set of challenges facing a population, whilst the adaptive response represents longer term trends above the population level. Observed patterns raise a number of potential questions for further study. The tools presented represent a significant advance in our ability to study the function of the immune system in a natural setting and allow the study of multiple replicate wild populations to be combined with controlled laboratory and field‐based infection experiments.

S.R., J.E.B. and A.D.C.M. designed the study and contributed to this manuscript. S.R. collected the samples and performed laboratory work and data analysis.

## Data accessibility

All qPCR expression and sampling data used in the analysis of wild and laboratory fish expression levels presented have been made publicly available, along with the expression culture data from the cell culture experiment, in the Dryad data repository, doi: 10.5061/dryad.3m2n4.

## Supporting information


**Appendix S1** (Supplementary Materials 1): Culture of stickleback cells.
**Appendix S2** (Supplementary Materials 2): Effect of sampling order on immune gene expression.
**Table S1** Summary of immune system genes targeted during primer design and development.
**Table S2** Summary of pairwise comparisons of immune gene expression levels of 8 genes, between wild caught and lab bred fish from two different populations (Reiv and Obse) and between lab bred fish only, using multivariate analysis of variance (manova).
