# Supplementary material for: Measuring the immune system of the three‐spined stickleback – investigating natural variation by quantifying immune expression in the laboratory and the wild
Source: Mol Ecol Resour. 2015 Dec 21;16(3):701–13. doi: 10.1111/1755-0998.12497 (PMC4991546; doi:10.1111/1755-0998.12497)
Supplement: Supplementary file 1 — Appendix S1 (Supplementary Materials 1): Culture of stickleback cells. Appendix S2 (Supplementary Materials 2): Effect of sampling order on immune gene expression. Table S1 Summary of immune system genes targeted during primer design and development. Table S2 Summary of pairwise comparisons of immune gene expression levels of 8 genes, between wild caught and lab bred fish from two different populations (Reiv and Obse) and between lab bred fish only, using multivariate analysis of variance (manova). [file MEN-16-701-s001.pdf]

Supplementary Materials 1

Culture of Stickleback Cells

For all steps in the protocol, RPMI 1640 culture media (Sigma-Aldrich R0883) was supplemented with 10% H_2O to adjust osmoregularity for fish cells. Work should be conducted in a sterile flow hood following standard sterile working procedures. The protocol has been tested on both whole spleens and head kidneys, and works on both tissue types. $RPMI^-$ is RPMI 1640 with 10% H_2O, whilst $RPMI^+$ is RPMI 1640 supplemented with 10% H_2O, 10% Foetal Bovine Serum, 2μM L-glutamine, 100μg/ml Penicillin and 100μg/ml Streptomycin.

Upon dissection of sampled fish, spleens and/or head kidneys should be placed immediately into ice cold $RPMI^-$ until ready to be prepared for culture.

The procedure to produce to create primary cell culture is as follows:

- Dissagregate cells by passing whole spleen or head kidneys through a 40μM cell strainer and suspend in 5ml of $RPMI^-$
- Centrifuge cell suspension for 2 minutes at 400rfc to pellet cells
- Pour off excess $RPMI^-$ and re-suspend cells. Top up with 4ml $RPMI^-$
- Repeat centrifugation and re-suspension step twice, using $RMPI^-$ for the first and $RPMI^+$ for the second.
- After final centrifugation, remove excess liquid with a pipette, and re-suspend cells in 400μl of $RPMI^+$

From the 400μl cell suspension, count the number of cells present in 20μl by staining with Trypan Blue. Adjust the concentration of cells in each sample to give 4×10^6 cells/ml. Cells should be cultured in a 100μl final volume, giving 40,000 cells/well.

Culture cells in 96-well flat bottomed cell culture plates. Add 75µl of cell suspension to 4 wells for each sample (2 control wells, 2 treatment wells). To the control wells, add 25µl additional $RPMI^+$. To the treatment wells, add 25µl of 50µg/ml Zymosan (Sigma-Aldrich Z4250).

Plates should be placed into an incubator at 20°C, with 5% CO_2 and saturated humidity.

For Zymosan stimulated cells, culture time should be approximately 24 hours, to allow an immune response to develop. Zymosan is a general antagonist of the immune system, here being used to promote an innate immune response. The use of other antagonists is possible, and combined with for longer culture times, allows for the activation of other parts of the immune system. Cells have been successfully cultured for up to 92 hours.

When removed from incubator, cells can be stored at -80°C until ready to extract RNA.

Supplementary Materials 2

Effect of Sampling Order on Immune Gene Expression

To ensure that the time which fish were held before processing and the order in which they were processed did not affect the expression of immune system genes, the correlation between sampling order and each of the immune groupings was calculated. Values were calculated using the ‘corr.test’ function of the ‘psych’ package in R v.3.1.2, with the Holm correction applied for multiple comparisons.

No significant correlations were found between sampling order and innate expression (r^2=-0.28, p=0.09), Th1-type expression (r^2=0.11, p=1), Th2-type expression (r^2=0.12, p=1), FoxP3a expression (r^2=0.07, p=1), and TGFβ expression (r^2=-0.11, p=1), as shown in figure S1.

Supplementary Table S1: Summary of immune system genes targeted during primer design and development.

Short Name	Full Name	Response	Sequence	ENSEMBL Gene ID	Amplification	Assay
IL-1B	Interleukin 1 Beta	proinflammatory	Full	ENSGACG00000014611	1	1
TNFa	Tumour Necrosis Factor Alpha	proinflammatory	Full	ENSGACG00000013372	1	1
NFκB	Nuclear Factor of Kappa Light Polypeptide gene enhancer in B cells	proinflammatory	Full**	ENSGACG00000016876	1	0
IL-10	Interleukin 10	Treg / regulatory	Partial		0	0
TGFB	Transforming Growth Factor Beta	Treg / regulatory	Full	ENSGACG00000012798	1	1
IFNγ	Interferon Gamma	Th1	Partial		1	0
IL-12	Interleukin 12	Th1	Full***	ENSGACG00000020455	0	0
IL-18	Interleukin 18	Th1	No			
Stat4	Signal Transducer and Activator of Transcription 4	Th1	Full	ENSGACG00000002684	1	1
T-bet	T box transcription Factor	Th1	Full	ENSGACG00000003829	1	1
IL-4	Interleukin 4	Th2	No			
IL-5	Interleukin 5	Th2	No			
IL-9	Interleukin 9	Th2	No			
IL-13	Interleukin 13	Th2	No			
Stat6	Signal Transducer and Activator of Transcription 6	Th2	Full	ENSGACG00000008477	1	1
CMIP	C-maf Inducing Protein	Th2	Full	ENSGACG00000002527	1	1
Gata3	Trans-acting T-cell-specific Transcription Factor GATA-3	Th2	Partial		0	0
IL-17	Interlukin 17	Th17	No			
IL-22	Interleukin 22	Th17	Partial		0	0
IL-6	Interleukin 6	Th17	Full	ENSGACG00000006102	1	0
ROR-γt	RAR-related Orphan Receptor Gamma t	Th17	Full	ENSGACG00000012239	1	0
IL-10	Interleukin 10	Treg / regulatory	Partial		0	0
TGFB	Transforming Growth Factor Beta	Treg / regulatory	Full	ENSGACG00000012798	1	1
FoxP3a	Forhead Box P3a	Treg / regulatory	Full	ENSGACG00000012777	1	1

‘Short Name’ and ‘Full Name’ refer to names used for genes in the ENSEMBL genome database. ‘Response’ indicated the arm of the immune response for which each gene is a marker. ‘Sequence’ denotes whether the gene was found in the stickleback genome, with ‘Full’ genes being named in the genome, and ‘Partial’ being found using homology to the gene sequence from another fish species, with corresponding ENSEMBL Gene ID numbers. ‘Amplification’ showed whether any primers amplified for a given gene, whilst ‘Assay’ indicated whether a fully working assay could be produced. ‘**’ denotes a gene where 2 copies where present in the genome, and ‘***’ a gene where three copies were present.

Supplementary Table S2: Summary of pairwise comparisons of immune gene expression levels of 8 genes, between wild caught and lab bred fish from two different populations (Reiv and Obse) and between lab bred fish only, using multivariate analysis of variance (MANOVA). Overall differences are shown, along with individual comparisons for each gene, calculated using the Pillia method. Significant p values (p<0.05) are indicated in bold.

	**Reiv Lab v Reiv Wild**			**Obse Lab v Obse Wild**			**Reiv Lab v Obse Lab**		
	F	df	p	F	df	p	F	df	p
Overall	11.31	20,1	**<0.001**	4.40	19,1	**0.011**	16.92	10,1	**0.020**
IL1B	6.51	20,1	**0.019**	0.72	19,1	0.406	28.47	10,1	**<0.001**
TNFa	13.13	20,1	**0.002**	2.30	19,1	0.146	10.95	10,1	**0.008**
Stat4	17.38	20,1	**<0.001**	15.67	19,1	**<0.001**	0.04	10,1	0.855
Tbet	73.45	20,1	**<0.001**	19.70	19,1	**<0.001**	0.01	10,1	0.919
Stat6	3.81	20,1	0.066	0.00	19,1	0.991	10.22	10,1	**0.009**
CMIP	24.23	20,1	**<0.001**	3.52	19,1	0.076	21.71	10,1	**<0.001**
TGFB	4.09	20,1	0.057	0.33	19,1	0.572	14.49	10,1	**0.003**
FoxP3	14.61	20,1	**0.001**	0.67	19,1	0.422	12.73	10,1	**0.005**
